# A multimodal artificial intelligence system for the detection and diagnosis of solid pancreatic lesions under EUS

**DOI:** 10.1097/eus.0000000000000145

**Published:** 2025-11-03

**Authors:** Chenxia Zhang, Xiao Tao, Jun Zhang, Wei Tan, Wei Zhou, Shan Hu, Bing Xiao, Honggang Yu

**Affiliations:** 1 Department of Gastroenterology, Renmin Hospital of Wuhan University, Wuhan, Hubei Province, China; 2 Hubei Provincial Clinical Research Center for Digestive Disease Minimally Invasive Incision, Renmin Hospital of Wuhan University, Wuhan, Hubei Province, China; 3 Key Laboratory of Hubei Province for Digestive System Disease, Renmin Hospital of Wuhan University, Wuhan, Hubei Province, China; 4 Taikang Center for Life and Medical Sciences, Wuhan University, Wuhan, Hubei Province, China; 5 National Engineering Research Center for Multimedia Software, School of Computer Science, Wuhan University, Wuhan, Hubei Province, China.

**Keywords:** Artificial intelligence, EUS, Solid pancreatic lesions

## Abstract

**Background and Objectives:**

Accurate differentiation of solid pancreatic lesions (SPLs) is crucial for treatment planning, but current methods still have limitations. Artificial intelligence (AI) has the potential to contribute to such diagnoses, yet existing AI models are restricted to focusing on a single modality. This study aims to develop a deep learning–based multimodal AI system to improve diagnostic accuracy for SPLs.

**Methods:**

A retrospective analysis was conducted on 492 patients who underwent EUS for SPLs at Renmin Hospital of Wuhan University between December 2016 and September 2024. The AI system consisted of four deep learning models: DCNN1 for focal pancreatic lesion detection, DCNN2 for classifying pancreatic lesions as cystic or solid, DCNN3 for lesion boundary segmentation and size measurement, and DCNN4 for classifying carcinoma and noncancerous lesions. For DCNN4, four different modality models were constructed: (1) model A: EUS B-mode images only. (2) model B: EUS-E images only. (3) model C: EUS B-mode images and EUS-E images. and (4) model D: EUS B-mode images, EUS-E images, and clinical data. The model performance was compared with the diagnostic performance of endoscopists.

**Results:**

The accuracy values of DCNN1 and DCNN2 were 96.8% and 98.9%, respectively. The Dice coefficient of the DCNN3 was 0.876. Our AI system demonstrated high accuracy, sensitivity, and specificity in differentiating carcinoma from noncancerous SPLs. The multimodal models, particularly those integrating EUS B-mode and EUS-E images, outperformed single-modality models, achieving an accuracy of 94.0% and an AUC of 0.937. The AI model showed superior performance compared to endoscopists, with improved diagnostic consistency and sensitivity.

**Conclusion:**

The multimodal AI system significantly improves the diagnostic accuracy of SPLs, providing a promising tool for the early detection and differentiation of pancreatic cancer.

## INTRODUCTION

Solid pancreatic lesions (SPLs) comprise a diverse group of pathological entities, among which pancreatic cancer is the most prevalent and aggressive, with a five-year survival rate of only 10%.^[1,2]^ Accurate preoperative differentiation of these lesions is crucial for determining optimal treatment strategies and improving patient prognosis. However, due to the deep anatomical location of the pancreas and its complex structural relationships with surrounding organs and vasculature, conventional imaging modalities such as computed tomography (CT) and magnetic resonance imaging (MRI) have limitations in both sensitivity and specificity for detecting early-stage tumors and distinguishing carcinoma from noncancerous lesions.^[3,4]^

EUS provides higher spatial resolution than CT and MRI.^[5,6]^ It enables fine-needle aspiration or biopsy (FNA/B) for histopathological diagnosis, making it an essential tool in the evaluation of pancreatic lesions.^[7]^ Despite these advantages, EUS performance is highly operator-dependent. Due to factors such as limited experience, operator fatigue, or distraction, this dependence on individual judgment can lead to missed diagnoses or incorrect evaluations.^[8]^ Studies indicate that the specificity of EUS in diagnosing malignant pancreatic lesions remains suboptimal, typically ranging from 50% to 60%.^[9]^ EUS elastography (EUS-E) has been introduced as a complementary technique to conventional B-mode EUS, providing additional diagnostic value without increasing procedural risk or duration.^[10]^ It offers information on tissue stiffness, which helps differentiate benign from malignant lesions.^[11]^ However, interpreting elastography remains challenging, as it often requires the operator to manually select the lesion area to obtain elasticity values. This reliance on subjective lesion selection and elasticity assessment can impact the overall diagnostic accuracy, highlighting the need for more objective and automated diagnostic approaches.

Recent advances in artificial intelligence (AI) demonstrate great potential in medical imaging, particularly in lesion detection, characterization, and risk stratification.^[12]^ AI-driven models can automate image analysis, minimizing human error and interobserver variability. However, most prior studies have focused on single-modality B-mode EUS imaging, which has inherent limitations.^[13–16]^ Because both noncancerous pancreatic lesions and carcinoma often present as solid masses with overlapping imaging features, relying solely on conventional B-mode EUS may not provide sufficient diagnostic precision.^[17]^ Moreover, B-mode EUS lacks standardization and quantifiable analysis, and its low interpretability may reduce clinician confidence. In clinical practice, endoscopists typically adopt a multimodal approach, integrating various imaging findings and clinical data to achieve a more comprehensive and reliable diagnosis.

Multimodal deep learning models, which leverage advanced AI architectures trained on diverse datasets, have emerged as a promising solution for enhancing lesion diagnosis.^[16,18,19]^ Unlike traditional single-modality approaches, multimodal AI systems allow for cross-modal feature extraction and analysis, substantially improving diagnostic performance.^[20,21]^ In this study, we developed a deep learning–based multimodal AI system to distinguish carcinoma from noncancerous SPLs under EUS. By integrating EUS B-mode images, EUS-E images, and structured clinical data, the system aims to improve diagnostic accuracy and assist clinical decision-making, ultimately optimizing the early detection and management of pancreatic cancer.

## PATIENTS AND METHODS

### Study design and patients

This retrospective study included patients who underwent EUS for pancreatic assessment at Renmin Hospital of Wuhan University between December 2016 and September 2024. The inclusion criteria were as follows: (1) age >18 years; (2) underwent EUS within 2 weeks before biopsy or surgery, with imaging findings indicative of SPLs; (3) had a confirmed pathological diagnosis or completed a minimum of 6 months of follow-up; and (4) had available EUS images and clinical data. The exclusion criteria were as follows: (1) a prior history of treatment for pancreatic lesions; (2) the presence of multiple pancreatic lesions; and (3) poor-quality EUS images, such as inadequate lesion visualization or image artifacts caused by the device. The study was approved by the Ethics Committee of Renmin Hospital of Wuhan University. Given its retrospective design, the requirement for written informed consent was waived.

### EUS and diagnostic procedure

EUS was performed using Olympus EU-ME1 and EU-ME2 (Olympus Medical Systems Co., Tokyo, Japan) or Fujifilm SU-1 (Fujinon Toshiba ES Systems Co., Ltd., Tokyo, Japan) processors with compatible endoscopes. All procedures were conducted by operators with at least 5 years of EUS experience. During the examination, the endoscopist first performed a comprehensive scan of the pancreas using conventional B-mode imaging until SPLs were identified. Subsequently, based on individual practice preferences, the operator could choose to activate Doppler mode or switch to elastography mode for further evaluation of the region of interest (ROI). The examination frequency for EUS was typically set at 7.5 MHz.

All SPLs were classified according to the following standard criteria: (1) SPLs with pathological diagnoses of pancreatic ductal adenocarcinoma (PDAC), pancreatic adenosquamous carcinoma (PASC), pancreatic acinar cell carcinoma (PACC), metastatic pancreatic tumors (MPT), and neuroendocrine carcinoma (NEC) were classified as carcinoma. (2) If no cancerous lesions were detected in specimens obtained from EUS-FNA/B and/or surgery, and no rapid disease progression in the pancreas was observed during the 6-month follow-up period, the lesion was classified as noncancerous. All histopathological assessments were conducted by pathologists with more than 10 years of experience in pancreatic pathology.

### System framework

**Figure 1:**
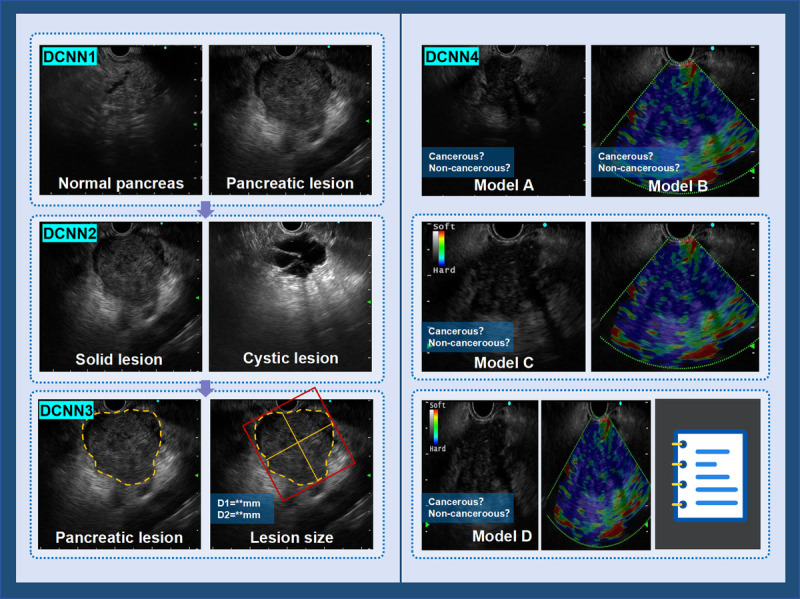
The framework of the multimodal artificial intelligence system.

The system is composed of two modules: lesion recognition and lesion diagnosis. The lesion recognition module employs DCNN1 to automatically detect focal pancreatic lesions in B-mode and activates the downstream lesion diagnosis module. The lesion diagnosis module consists of three deep learning models, each tasked with a specific function. First, DCNN2 classifies the detected lesions as cystic or solid and filters out cystic pancreatic lesion images. Then, DCNN3 segments the boundaries of the SPLs and automatically measures the size of the lesions. Finally, DCNN4 further differentiates between cancerous and noncancerous lesions in the SPLs. The overall workflow of the system is shown in Figure 1.

### Deep convolutional neural network development

We employed ResNet for image classification and Unet++ for image segmentation. Images from the same patient were not included in both the training and test sets. A total of 1907 normal pancreatic images and 2027 pancreatic lesion images were used to train and test DCNN1 for the detection of pancreatic lesions under EUS. During the lesion diagnosis stage, DCNN2 was trained and tested on 1007 cystic pancreatic lesion images and 1020 solid pancreatic lesion images. DCNN3 was trained and tested on 3853 pancreatic lesion images to label lesion boundaries. The above images were all EUS B-mode images. All images were annotated by two experts with over 10 years of EUS experience (Expert A and Expert B), whose annotations served as the gold standard for the construction of the aforementioned models. Detailed information on the construction process for each model is provided in Appendix S1, http://links.lww.com/ENUS/A386.

For the classification of cancerous and noncancerous lesions (DCNN4), we constructed four different modality models: (1) model A: EUS B-mode images only; (2) model B: EUS-E images only; (3) model C: EUS B-mode images and EUS-E images; and (4) model D: EUS B-mode images, EUS-E images, and clinical data (age, sex, CEA, CA199, CA125, amylase, lipase). Because elastography is not routinely enabled at our center, only a subset of patients have EUS-E image data. To compare the performance of the different models, we divided the patients into two groups. The test set consisted of SPL patients with elastography data from June 2022 to September 2024, whereas the remaining patients were used for the training set. The dataset division for each model is shown in Table S1 (http://links.lww.com/ENUS/A386). Detailed information on the construction process for models A–D is provided in Appendix S2, http://links.lww.com/ENUS/A386.

### Man-machine comparison

The EUS B-mode images, EUS-E images, and clinical data of SPL patients with elastography data from June 2022 to September 2024 were used as the dataset for the man-machine comparison. Eight endoscopists from Renmin Hospital of Wuhan University were invited, including three junior endoscopists (EUS experience <5 years), three senior endoscopists (EUS experience 5–10 years), and two expert endoscopists (EUS experience >10 years). In the diagnostic process, for each patient, the endoscopist initially made a diagnosis of cancer or non–cancer-based solely on the EUS B-mode images. Then, the EUS-E images of the lesion were provided, and the endoscopist was asked to make a second diagnosis based on these images. Finally, the patient’s clinical information was provided, and the endoscopist was asked to make the final diagnosis by combining the image data with the clinical information. Each diagnostic stage had to be completed independently, and the results from previous stages were not allowed to be modified.

### Statistical analysis

Normally distributed continuous variables were presented as mean ± standard deviation (SD), whereas non-normally distributed data are expressed as median with interquartile range (IQR). Comparisons between the two groups were conducted using the *t* test or Mann-Whitney *U* test. Categorical variables were described as frequency (percentage), and intergroup comparisons were made using the *χ*^2^ test or Fisher’s exact test. For classification models, the model performance was evaluated by calculating overall accuracy, sensitivity, specificity, positive predictive value (PPV), negative predictive value (NPV), and the area under the receiver operating characteristic curve (AUC). For segmentation models, performance was assessed using precision, recall, and Dice coefficient. Interobserver agreement of the endoscopists and the model was evaluated using Cohen’s kappa coefficient. *P* < 0.05 was considered statistically significant. All statistical analyses were conducted using SPSS 25 (IBM, Chicago, IL, USA).

## RESULTS

### Patient characteristics

**Figure 2:**
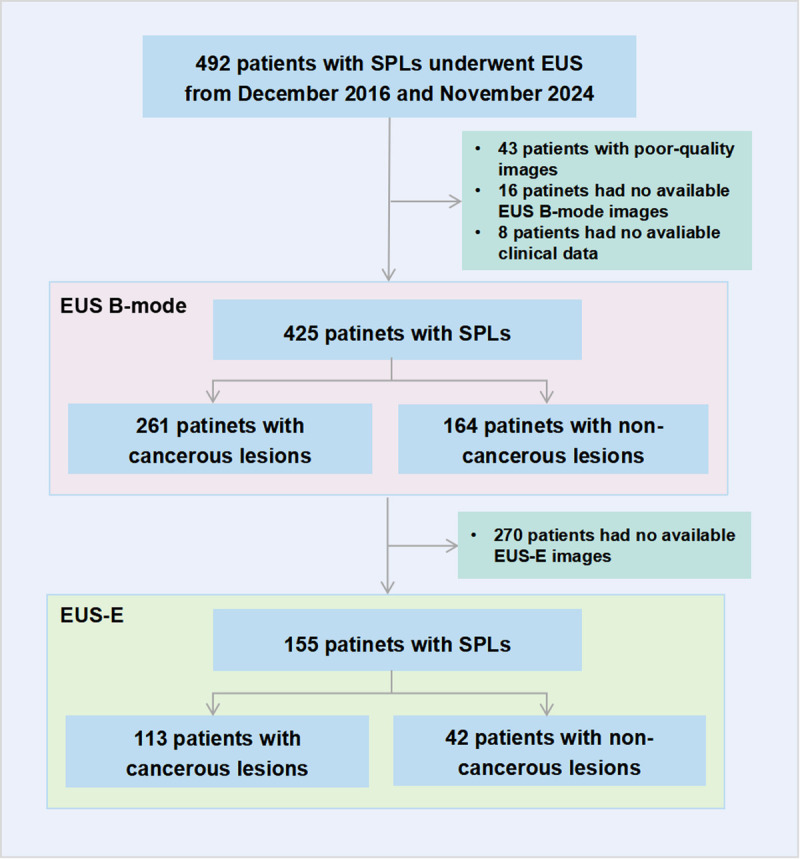
Flowchart of the study population.

As shown in Figure 2, data from 492 patients who underwent EUS procedures for SPLs were retrospectively collected from Renmin Hospital of Wuhan University between December 2016 and September 2024. After excluding patients with poor-quality images (*n* = 43) and missing data (*n* = 24), 425 patients were included in the study. Among them, 261 had cancerous lesions, whereas 164 had noncancerous lesions. A total of 155 patients had available EUS-E images, including 113 with cancerous lesions and 42 with noncancerous lesions. Table 1 presents the baseline characteristics of patients in the EUS B-mode and EUS-E datasets. Among patients with carcinoma, the average age was 63.6 ± 9.0 years, with 151 male patients (57.9%). A total of 351 (82.6%) patients had lesions larger than 2 cm, and the lesions in the noncancerous group were significantly smaller than those in the cancerous group. More than half of the SPLs were located in the pancreatic head and neck.

**Table 1:** Patient demographics and characteristics of pancreatic lesions.

Characteristics	EUS B-mode dataset (*n* = 425)			EUS-E dataset (*n* = 155)		
	Cancerous (*n* = 261)	Noncancerous (*n* = 164)	*P* value	Cancerous (*n* = 113)	Noncancerous (*n* = 42)	*P* value
Age (yr), mean ± SD)	63.6 ± 9.0	56.4 ± 12.6	<0.001	65.1 ± 9.0	53.5 ± 14.1	0.002
Sex			0.524			0.800
Male	151 (57.9)	100 (61.0)		62 (54.9)	24 (57.1)	
Female	110 (42.1)	64 (39.0)		51 (45.1)	18 (42.9)	
Lesion size			<0.001			0.001
≤2 cm	26 (10.0)	48 (29.3)		8 (7.1)	11 (26.2)	
>2 cm	235 (90.0)	116 (70.7)		105 (92.9)	31 (73.8)	
Lesion location			0.707			0.121
Head or neck	156 (59.8)	95 (57.9)		72 (63.7)	21 (50.0)	
Body or tail	105 (40.2)	69 (42.1)		41 (36.3)	21 (50.0)	
Laboratory data						
Serum CEA (ng/mL), median (IQR)	3.04 (1.64–7.79)	1.45 (0.83–2.42)	<0.001	2.35 (1.48–5.94)	0.96 (0.50–1.88)	<0.001
Serum CA125 (U/mL), median (IQR)	29.10 (10.48–107.30)	11.20 (7.50–20.80)	<0.001	17.10 (8.85–80.70)	8.50 (7.20–15.00)	0.008
Serum CA199 (U/mL), median (IQR)	360.82 (29.44–1505.66)	19.39 (8.79–37.75)	<0.001	348.18 (25.08–1637.44)	14.07 (7.03–28.96)	<0.001
Serum amylase (U/L), median (IQR)	57.00 (43.00–87.00)	70.00 (51.00–131.00)	0.003	52.00 (43.00–86.00)	62.00 (48.00–98.00)	0.255
Serum lipase (U/L), median (IQR)	182.50 (70.25–491.25)	198.50 (111.00–681.50)	0.097	142.00 (69.00–477.00)	162.00 (111.5–530.00)	0.597

### Model performance

The DCNN1 model had a 96.8% accuracy in classifying normal pancreatic images and pancreatic lesion images, with a sensitivity of 99.6% and a specificity of 93.6% for lesion identification [Figure S1, http://links.lww.com/ENUS/A386]. The accuracy of DCNN2 to distinguish cystic and solid pancreatic lesion EUS images was 98.9%, with a sensitivity of 99.6% and a specificity of 98.3% for SPL identification [Figure S2, http://links.lww.com/ENUS/A386]. The Dice coefficient for the segmentation model (DCNN3) was 0.876, with a recall and precision of 87.1% and 88.1%, respectively, at a 50% intersection over union (IoU) threshold. The intraclass correlation coefficient (ICC) between AI-based lesion size measurements and measurements by endoscopists was 0.827.

**Figure 3:**
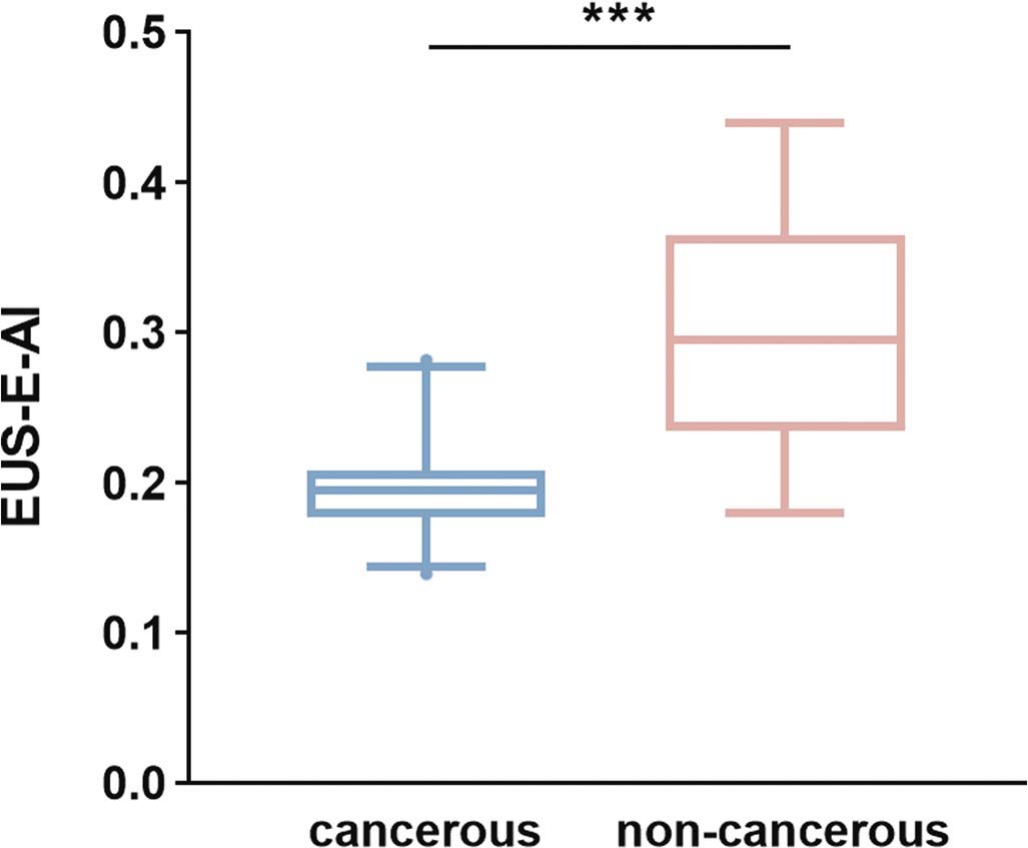
The EUS-E-AI values of SPLs patients. ***P < 0.001.

In the test set of 67 cases (52 carcinoma, 15 noncarcinoma), the classification performance of models A–D is summarized in Table 2. Model A, which relied solely on EUS B-mode images, demonstrated the poorest performance, achieving an accuracy of 70.2% and AUC of 0.749 in distinguishing carcinoma from noncancerous lesions. For model B, we extracted elasticity values (EUS-E-AI) from the lesion regions in EUS-E images using an AI-based approach, as detailed in the model construction section of the Appendix S2, http://links.lww.com/ENUS/A386. The median EUS-E-AI value in the cancerous group (0.195 [IQR, 0.177–0.208]) was significantly lower than that in the noncancerous group (0.295 [IQR, 0.234–0.365], *P* < 0.001) [Figure 3]. In the receiver operating characteristic analysis, the optimal cutoff value of EUS-E-AI for differentiating carcinoma from noncancerous lesions was 0.223. At this threshold, the sensitivity, specificity, PPV, NPV, accuracy, and AUC were 88.5%, 80.0%, 93.9%, 66.7%, 86.6%, and 0.910, respectively. Compared to the single-modality models A and B, the multimodal models C and D exhibited improved performance. Among them, the model integrating EUS B-mode and EUS-E images achieved the highest classification accuracy of 94.0% and an AUC of 0.937.

**Table 2:** Diagnostic performance of models A–D.

	Accuracy (95% CI)	Sensitivity (95% CI)	Specificity (95% CI)	PPV (95% CI)	NPV (95% CI)
Model A	70.2 (59.2–81.1)	71.2 (58.8–83.5)	66.7 (42.8–90.5)	88.1 (78.3–97.9)	40.0 (20.8–59.2)
Model B	86.6 (78.4–94.4)	88.5 (79.8–97.2)	80.0 (59.8–100.0)	93.9 (87.2–100.0)	66.7 (44.9–88.4)
Model C	94.0 (88.4–99.7)	98.1 (94.3–100.0)	80.0 (59.8–100.0)	94.4 (88.3–100.0)	92.3 (77.8–100.0)
Model D	92.5 (86.5–98.8)	96.2 (90.9–100.0)	80.0 (59.8–100.0)	94.3 (88.1–100.0)	85.7 (67.4–100.0)

### Comparison between models and endoscopists

We conducted a man-machine contest among model A, model C, model D, and eight endoscopists on the test set. For EUS B-mode single-modal data, the accuracy (70.2% *vs.* 61.9%, *P* = 0.006), sensitivity (71.2% *vs.* 61.5%, *P* = 0.004), and specificity (66.7% *vs.* 63.3%, *P* = 0.694) of model A were better than the average of eight endoscopists. For multimodal data, model C and model D performed better than the average of endoscopists in accuracy (model C: 94.0% *vs.* 71.0%, *P* < 0.001; model D: 92.5% *vs.* 74.1%, *P* < 0.001) and sensitivity (model C: 98.1% *vs.* 72.6%, *P* < 0.001; model D: 80.0% *vs.* 73.3%, *P* < 0.001), with a comparable specificity (model C: 80.0% *vs.* 65.0%, *P* = 0.010; model D: 80.00% *vs.* 76.7%, *P* = 0.596). Either endoscopists or the machine performed better when multimodal data were available. AI achieves better results than endoscopists in both single-modal and multimodal modes. The performances of individual endoscopists are reported in Table 3. The interobserver agreement between models A, C, and D and the experts is shown in Table 4. Multimodal models C and D demonstrated strong agreement (kappa scores of 0.954), with higher consistency with the two experts compared to the single-modal model.

**Table 3:** Diagnostic performance of endoscopists.

			EUS B-mode			EUS B-mode and EUS-E			EUS B-mode and EUS-E & Clinical data		
			Accuracy	Sensitivity	Specificity	Accuracy	Sensitivity	Specificity	Accuracy	Sensitivity	Specificity
Average of total		61.9 (57.8–66.1)	61.5 (56.9–66.2)	63.3 (54.7–72.0)	71.0 (67.1–74.7)	72.6 (68.3–76.9)	65.0 (56.5–73.5)	74.1 (70.4–77.8)	73.3 (69.1–77.6)	76.7 (69.1–84.2)
Average of non-expert		55.0 (50.1–59.8)	52.2 (46.7–57.8)	64.4 (54.6–74.3)	65.2 (60.5–69.8)	66.4 (61.1–71.6)	61.1 (51.0–71.2)	68.7 (64.1–73.2)	66.4 (61.1–71.6)	76.7 (67.9–85.4)
Average of expert		82.8 (76.5–89.2)	89.4 (83.5–95.3)	60.0 (42.5–77.5)	88.1 (82.6–93.6)	91.4 (85.9–96.8)	76.7 (61.5–91.8)	90.3 (85.3–95.3)	94.2 (88.0–97.4)	76.7 (61.5–91.8)
Junior	Endoscopist 1	47.8 (35.8–59.7)	44.2 (30.7–57.7)	60.0 (35.2–84.8)	50.1 (38.8–62.7)	51.9 (38.3–65.5)	46.7 (21.4–71.9)	55.2 (43.3–67.1)	53.9 (40.3–67.4)	60.0 (35.2–84.8)
	Endoscopist 2	52.2 (40.3–64.2)	50.0 (36.4–63.6)	60.0 (35.2–84.8)	62.7 (51.1–74.3)	63.5 (50.4–76.6)	60.0 (35.2–84.8)	70.2 (59.2–81.1)	65.4 (52.5–78.3)	86.7 (69.5–100.0)
	Endoscopist 3	50.8 (38.8–62.7)	46.2 (32.6–59.7)	66.7 (42.8–90.5)	62.7 (51.1–74.3)	63.5 (50.4–76.6)	60.0 (35.2–84.8)	73.1 (62.5–83.6)	73.1 (61.0–85.1)	73.3 (50.1–95.7)
Senior	Endoscopist 4	55.2 (43.3–67.1)	53.9 (40.3–67.4)	60.0 (35.2–84.5)	70.2 (59.2–81.1)	76.9 (65.5–88.4)	46.7 (21.4–71.9)	68.7 (57.6–79.8)	65.4 (52.5–78.3)	80.0 (59.8–100.0)
	Endoscopist 5	59.7 (48.0–71.5)	50.0 (36.4–63.6)	93.3 (80.7–100.0)	70.2 (59.2–81.1)	63.5 (50.4–76.6)	93.3 (80.7–100.0)	68.7 (57.6–79.8)	61.5 (48.3–74.8)	93.3 (80.7–100.0)
	Endoscopist 6	64.2 (52.7–75.7)	69.2 (56.7–81.8)	46.7 (21.4–72.0)	74.6 (78.9–67.8-90.0)	76.1 (65.9–86.3)	78.9 (67.8–90.0)	76.1 (65.9–86.3)	78.9 (67.8–90.0)	66.7 (42.8–90.5)
Expert	Endoscopist 7	86.6 (78.4–94.7)	94.2 (87.9–100.0)	60.0 (35.2–84.8)	94.0 (88.4–99.7)	98.1 (94.4–100.0)	80.0 (59.8–100.0)	94.0 (88.4–99.7)	98.1 (94.3–100.0)	80.0 (59.8–100.0)
	Endoscopist 8	79.1 (69.4–88.8)	84.6 (74.8–94.4)	60.0 (35.2–84.8)	82.1 (72.9–91.3)	84.6 (74.8–94.4)	73.3 (50.1–95.7)	86.6 (78.4–94.7)	90.4 (82.4–98.4)	73.3 (50.1–95.7)

**Table 4:** Interobserver agreement (kappa value) of experts and models A, C, and D.

	EUS B-mode			EUS B-mode and EUS-E			EUS B-mode and EUS-E and Clinical data		
	Endoscopist 7	Endoscopist 8	Model A	Endoscopist 7	Endoscopist 8	Model C	Endoscopist 7	Endoscopist 8	Model D
Endoscopist 7									
Endoscopist 8	0.520			0.513			0.605		
Model A	0.215	0.249		0.223	0.329	0.364	0.223	0.415	0.405
Model C	0.558	0.402	0.364	0.618	0.513		0.618	0.605	0.954
Model D	0.523	0.372	0.405	0.583	0.481	0.954	0.583	0.571	

## DISCUSSION

With the widespread application of EUS in diagnosing biliopancreatic diseases, an increasing number of SPLs are being identified. These lesions exhibit significant variations in treatment according to their pathological types, highlighting the importance of accurate classification. Although EUS is considered the preferred method for evaluating focal pancreatic lesions, its results are highly dependent on the operator’s expertise, leading to low diagnostic accuracy. AI offers a promising solution to improve the diagnostic performance of EUS for pancreatic lesions. This study proposes an AI system based on multimodal data to identify and diagnose pancreatic lesions under EUS, accurately distinguishing between cancerous and noncancerous SPLs. The diagnostic performance of our system was compared with the results of endoscopists, and the results showed that the AI model outperformed human diagnoses in accuracy, achieving high concordance with expert assessments.

In recent years, several studies have explored the application of AI in CT, MRI, and EUS for the diagnosis of SPLs.^[22,23]^ Cao et al. developed a deep learning model based on non–contrast-enhanced CT, achieving an AUC of 0.986. Compared to the average performance of radiologists, their model demonstrated higher sensitivity and specificity in identifying PDAC, with improvements of 34.1% and 6.3%, respectively.^[24]^ Kuwahara et al. conducted a retrospective analysis of EUS B-mode images and developed an AI-based model to differentiate pancreatic cancer from noncancerous lesions with reliable diagnostic performance.^[25]^ Despite promising results, these studies predominantly rely on single-modality imaging data, neglecting the complexities in clinical workflows. In real-world clinical practice, diagnostic decisions are typically based on a comprehensive analysis of various sources of information, including imaging data and clinical data.^[26,27]^ Single-modal models may overly rely on certain specific features; this bias can impair the model’s flexibility and comprehensiveness, particularly when dealing with complex or heterogeneous lesions. In the man-machine comparison, we found that the diagnostic consistency among endoscopists was poor when only EUS B-mode images were provided. Even experts saw significant improvements in diagnostic accuracy when additional clinical information was included. Thus, multimodal models that combine EUS images with clinical data are better aligned with clinical needs and have the potential to be more effectively integrated into clinical practice.

Cui et al. constructed a multimodal “Joint-AI” model by combining clinical data from 439 SPL patients with EUS B-mode images.^[27]^ This multimodal model significantly enhanced diagnostic efficacy compared to single-modality models based only on EUS B-mode images, further validating the effectiveness of multimodal data in pancreatic lesion diagnosis. However, the study incorporated 36 clinical features, including CT attenuation in the pancreatic parenchymal phase, MRI T1-weighted signal, and MRI T2-weighted signal, which increased the model’s complexity, limiting its application in routine clinical practice. In addition, previous studies based on EUS mostly utilized only EUS B-mode images.^[25,28]^ Elastography serves as a valuable supplement to B-mode, enabling real-time assessment of tissue stiffness.^[10]^ Malignant tumors, particularly those associated with fibrosis or infiltrative lesions, typically exhibit increased stiffness. Currently, the synergistic effects between EUS-E and other modality data, as well as the diagnostic performance of different models, have yet to be fully compared and evaluated.

Previous studies have evaluated the effectiveness of EUS-E in differentiating between benign and malignant pancreatic lesions.^[29]^ In EUS-E images, green regions typically correspond to soft tissue and are considered benign, whereas blue regions are more likely to indicate malignancy. Itokawa et al. conducted a retrospective analysis of 109 patients with SPLs and found that all pancreatic cancers appeared deep blue in EUS-E images, whereas inflammatory masses presented as green, yellow, or light blue.^[30]^ To address the bias introduced by visual color assessment, strain ratio (SR) was subsequently employed for semiquantitative analysis of pancreatic lesions.^[31,32]^ This method requires the endoscopist to select a circular ROI over the lesion (A) and another smaller ROI (B) over a soft reference area to calculate SR.^[33]^ Although this approach reduces the bias in color evaluation, it still involves some subjectivity, as the ROI selection remains operator-dependent. Moreover, the reference area is typically chosen from the softest part of the gastric wall, but variations in the size and position of this reference area relative to the lesion can significantly impact measurement accuracy.^[11]^ Several studies have measured the average SR value of malignant tumors and explored diagnostic thresholds, but due to unavoidable human bias, the optimal diagnostic threshold has yet to be determined.^[32,34]^ Minimizing interobserver variability and improving the reproducibility of diagnostic results continue to be major challenges for EUS-E.

In this study, deep learning–based lesion classification and segmentation models were developed for the automatic identification and boundary segmentation of SPLs, eliminating the need for manual ROI selection by the operator. This overcomes the limitations of traditional EUS scanning methods and reduces bias related to ROI selection. On this basis, we proposed a new quantitative index, EUS-E-AI, calculated by averaging the pixel values of all colored pixels within the segmented lesion region on elastography images. This method provides a reproducible and objective measure of tissue stiffness that is independent of operator experience and visual interpretation, and demonstrated favorable diagnostic performance with an AUC of 0.910. However, it is worth noting that calculating the average stiffness over the entire lesion may not fully reflect the internal heterogeneity of pancreatic tumors. Malignant lesions often exhibit regional variation in stiffness, and in some cases, such as tumors with central necrosis or infiltrative growth patterns, localized high-stiffness regions may hold stronger diagnostic value than global averages. To address this limitation, we plan to further refine our elastography assessment strategy by subdividing the segmented lesion into multiple subregions (“elasticity microzones”) and calculating stiffness values for each zone independently. These localized stiffness values will be evaluated against our previously established diagnostic thresholds to identify high-risk areas within the lesion. This approach may enhance the sensitivity of cancer detection and guide EUS-FNA/B toward more representative regions, thereby improving the diagnostic yield of tissue sampling.

To further improve diagnostic accuracy, we developed single-modality and multimodal AI models by integrating EUS B-mode images, EUS-E images, and clinical data, and compared their performance. The results revealed that the multimodal model combining EUS B-mode and EUS-E images significantly outperforms the single-modal model that relied solely on EUS B-mode images. With the inclusion of multimodal information, the results from the expert and AI model exhibit strong consistency. This finding highlights the advantages of multimodal data fusion, enabling a more comprehensive extraction and analysis of lesion features. More importantly, the data used in this study, including EUS-E images and laboratory markers, are easily accessible, making this approach highly feasible for integration into routine clinical workflows.

The diagnosis of pancreatic cancer remains a significant clinical challenge, especially when EUS-FNA/B results are negative despite strong clinical suspicion. The multimodal AI system developed in this study provides reliable supplementary information to EUS-FNA/B, assisting clinicians in making more informed decisions in complex situations where pathological results are uncertain. Meta-analyses have demonstrated that the sensitivity and NPV of EUS-FNA/B in diagnosing pancreatic cancer are 85%–89% and 45%–75%, respectively.^[35,36]^ Although a positive pathological diagnosis is not obligatory before surgery, obtaining a definitive diagnosis is crucial to avoid unnecessary procedures and the risk of removing benign lesions.^[37]^ In cases with negative EUS-FNA/B results, supplementary techniques such as genetic mutation analysis and repeated EUS-FNA/B are usually considered, which impose additional invasive procedures and financial burdens on patients.^[38]^ In this context, our multimodal AI model achieved a sensitivity of 94.0% and an NPV of 92.3% in identifying pancreatic cancer, demonstrating its strong diagnostic capability. This is particularly valuable for patients where the model suggests cancer, but the EUS-FNA/B biopsy results are negative, as it prompts endoscopists to intervene promptly and reduce the risk of missed diagnoses.

To further assess the system’s performance in this clinically relevant context, we conducted a subgroup analysis of 17 patients in the test cohort who had negative EUS-FNA/B results but ultimately received definitive diagnoses through surgery or long-term follow-up. Among them, 4 patients were confirmed to have pancreatic cancer and 13 had benign lesions. When evaluated using our multimodal AI model, the system correctly identified all 4 malignant cases and 11 of the 13 benign cases, yielding a sensitivity of 100% and an NPV of 100% in this subgroup. Although the number of patients in this subgroup was limited, these findings are promising and support the potential role of the AI system as a supportive tool in cases with inconclusive cytology, helping guide further clinical decisions such as repeat sampling or close surveillance, and potentially minimizing diagnostic delays in high-risk patients.

Several limitations of this study should be acknowledged. First, the study utilized single-center data from the Renmin Hospital of Wuhan University, which may introduce potential biases. Therefore, prospective multicenter studies are required to improve the generalizability of the model and validate its performance in diverse clinical settings. Second, because elastography was not routinely performed by endoscopists at our center, the test set for each model was limited to patients with EUS-E data available from June 2022 to September 2024. To further evaluate the additional value of EUS-E images, future prospective studies should routinely incorporate elastography and collect a larger dataset of such images.

In conclusion, we developed a multimodal AI system capable of identifying and diagnosing SPLs under EUS. By integrating EUS B-mode images, EUS-E images, and clinical data, the system demonstrates robust performance in detecting and diagnosing pancreatic lesions. This tool has the potential to assist in differential diagnosis, treatment decision-making, and follow-up planning for patients with SPLs.

## Acknowledgment

None.

## Source of Funding

This study was funded by the Key Research and Development Program of Hubei Province (grant no. 2023BCB153, to Honggang Yu) and the Project of Hubei Provincial Clinical Research Center for Digestive Disease Minimally Invasive Incision (grant no. 2024CCB007, to Honggang Yu).

## Ethical Approval

The study was approved by the Ethics Committee of Renmin Hospital of Wuhan University.

## Informed Consent

Given its retrospective design, the requirement for written informed consent was waived.

## Conflicts of Interest

All authors declare no conflicts of interest.

## Authors Contributions

H. Yu conceived and designed the study; C. Zhang and X. Tao conducted the data collection and verification; C. Zhang, X. Tao, J. Zhang, W. Tan, W. Zhou, S. Hu, and B. Xiao contributed to the statistical analysis and the development of the methodology used for data interpretation; C. Zhang and X. Tao drafted the manuscript, whereas J. Zhang, W. Tan, W. Zhou, S. Hu, and B. Xiao contributed to revising and refining the manuscript; all authors reviewed and approved the final manuscript for submission.

## Data Availability Statement

The data that support the findings of this study after deidentification are available from the corresponding author upon reasonable request.
